# Quality use of medicines within universal health coverage: challenges and opportunities

**DOI:** 10.1186/1472-6963-14-357

**Published:** 2014-08-27

**Authors:** Anita K Wagner, Jonathan D Quick, Dennis Ross-Degnan

**Affiliations:** Department of Population Medicine, Harvard Medical School and Harvard Pilgrim Health Care Institute, 133 Brookline Avenue, Boston, MA 02115 USA; Management Sciences for Health, 200 Rivers Edge Drive, Medford, MA 02155 USA

**Keywords:** Universal health coverage, Medicines, Pharmaceutical benefit, Financial incentives, Health systems, Quality of care

## Abstract

**Background:**

Medicines are a major driver of quality, safety, equity, and cost of care in low and middle-income country health systems. Universal health coverage implementers must explicitly address appropriate use of medicines to realize the health benefits of medicines, avoid wasting scarce resources, and sustain the financial viability of universal health coverage schemes.

**Discussion:**

Medicines are major contributors to the health and well-being of individuals and populations when used appropriately, and they waste resources and endanger health when used unnecessarily or incorrectly. Stakeholders need to balance inherently competing objectives in the pharmaceutical sector. Emerging and expanding UHC schemes provide potential levers to balance competing system objectives.

To use these levers, sustainable universal coverage programs will require a) information systems that can track medicines utilization, expenditures, and quality of medicines use; b) routine monitoring of indicators of medicines availability, access, affordability, and use; c) policies and programs that facilitate appropriate medicines use by prescribers, dispensers, and patients; d) transparency in setting priorities for medicines coverage under resource constraints; and e) a system perspective to engage diverse actors.

As they operationalize paths toward universal health coverage and include targeted medicines coverage policies and programs, systems can build on, and innovate, pharmaceutical policy frameworks and management tools from different countries’ settings.

**Summary:**

Ensuring that medicines which achieve important health outcomes are available, accessible to all, used appropriately, and sustainably affordable is essential for realizing universal health coverage. Stakeholder cooperation and use of information and financing system levers provide opportunities to work toward this goal.

## Background

On August 15, 2013, WHO Director General Dr. Chan introduced the World Health Report 2013 saying “Universal coverage means quality health care for all delivered in ways that protect users from financial ruin or impoverishment” and highlighted that “[T] he challenge is to expand health services with constant attention to causes of waste and inefficiency that can be reduced through smart policies and wise decisions [1].”

We argue that UHC implementers must explicitly focus on medicines, which are one of the major drivers of quality, safety, equity, and cost of care in low and middle-income country (LMIC) health systems. We first provide a brief overview of the typical medicines situation in LMICs; second, we describe the competing objectives of pharmaceutical sector policies and suggest that systems striving toward UHC have unique levers at their disposal to balance these objectives; and third, we highlight selected tools and approaches that systems working toward UHC should consider when developing targeted medicines policies and programs. We conclude with key challenges that systems will face as they move toward evidence-informed medicines coverage policies.

## Discussion

### Medicines and UHC - needs and opportunities

#### Medicines in health systems

Medicines are major contributors to the health and well-being of individuals and populations when used appropriately, and they waste resources and endanger health when used unnecessarily or incorrectly. Global medicines spending has surpassed US $1 trillion per year [2] and accounts for up to 67% of total health expenditures in some countries [3], mostly paid out of pocket by consumers. At the same time, medicines constitute three of the top ten sources of waste of scarce health system resources [4], due, among other factors, to underuse of quality generic products [5]; taxes and tariffs increasing product prices at different levels [6]; unreliable availability of medicines in public sector facilities; [7] substandard and falsified products in markets [8]; and inappropriate use of medicines, including overuse of antibiotics (often for children with respiratory infection or diarrhea) [9] and underuse of proven therapies for chronic conditions (e.g., hypertension, diabetes) [10, 11]. Meanwhile, households face impoverishment due to paying for medicines [12, 13] while patients die prematurely because they lack access to lifesaving medicines [14]. Providing access to novel high-cost specialty medicines for prevalent chronic conditions like cancers poses a growing ethical and economic challenge for policy makers in countries at all income levels.

### Competing objectives of medicines policies

**Figure 1:**
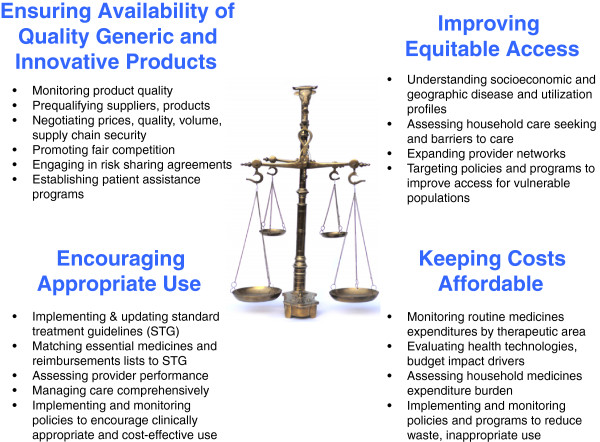
**Competing objectives in the medicines sector and selected approaches to balance them.**

Stakeholders need to strike a balance between several competing objectives in the pharmaceutical sector (Figure 1). Ideally, all patients, particularly vulnerable ones, would have access to the medicines they need according to evidence-based treatment guidelines; products would be of proven quality; appropriately prescribed medicines would be available where and when patients need them; and patients would take these medicines as needed to achieve their clinical effects. In this ideal world, both households and the health system would have the resources to pay for necessary medicines, in light of competing demands. To meet national concerns for a strong economy and health security, local manufacturers would be profitable while providing high-quality products at costs that patients and systems could afford. Research-based companies would develop innovative products for unmet needs. All stakeholders would adhere to transparent governance and ethical business practices. However, these idealized pharmaceutical sector objectives inevitably compete in many ways. For example, paying for medicines for people who cannot afford them may exceed the resources of systems. Limiting third party payments to preserve the financial sustainability of health systems may increase out-of-pocket household spending for medicines and limit appropriate use [15].

### Potential UHC levers to balance objectives

Emerging and expanding UHC schemes have potential levers to balance competing system objectives. Because they enroll members and pay for their care, schemes could access information on the demographic characteristics, health care needs, and utilization patterns of members. Because they employ or contract with providers, UHC schemes can also know about the demographic characteristics, prescribing patterns, and costs associated with specific providers. In their role as financial intermediaries, UHC schemes have leverage to determine what types of care they pay for and how much they will pay, and they can implement policies that provide incentives for purchasing, prescribing, and using the most clinically appropriate and safe medicines that are known to achieve highly valued health outcomes. Since they pay for large quantities of medicines, UHC schemes are also in a position to negotiate product prices, dictate standards of product quality, react to unethical promotion practices, and demand supply channel efficiency.

Practically, most LMIC insurance schemes do not yet use many of these levers [16, 17]. Some schemes may not know the demographics and clinical needs of their covered population. Schemes often have inefficient claims processing systems, some based on paper forms, which may make it difficult to access information about medicines utilization in a timely way. Often, schemes limit claims review primarily to detecting instances of fraud. Working actively with members, providers, and manufacturers to set performance standards and shape patterns of pharmaceutical care is currently beyond the scope or capacity of most UHC systems. Historical experience [18] indicates that schemes tend to focus explicitly on medicines only when medicines expenditures threaten the viability of the scheme, and they then focus primarily on prices rather than quality medicines use.

### Targeted medicines benefit strategies

We believe that not every available medicine for every individual could or should be subsidized by a third party payer. Targeted approaches to promote clinically appropriate, cost-effective medicines use require systems to understand populations, clinical conditions, medicines, settings for safe use, costs, and system capacity. Depending on their values, goals, and resources, UHC systems may choose to cover only specific medicines for defined conditions in specific population groups, treated at the lowest system level at which safe and effective care can be provided, and at costs that take into account members’ and the system’s ability to pay.

Tools and approaches already exist that UHC schemes in LMIC can adapt for use in their settings (Figure 1). International [19] and local data on population disease epidemiology, combined with information on current patterns of care, can help to identify the most important health conditions for which medicines are needed in a given population. Given a set of priority health conditions, evidence-based clinical guidelines [20] can be used to determine the types of medicines needed, which provide a basis for medicine reimbursement lists. Health technology assessment, budget impact analysis, and frameworks for transparency can inform ethically challenging decisions [21] about setting limits on covered therapies. Participation in international product quality assurance collaborations [22] can help ensure product quality, while strengthening capacity for managing medicines in systems [23] can improve the efficiency and reliability of supply. Widely-used facility-based indicators of appropriate and inappropriate medicines use in LMIC [9] could be adapted for use with insurance claims data and expanded to assess medicines use and expenditures for specific conditions.

To ensure financial viability, improve efficiency, and facilitate appropriate use, LMIC insurance systems need to pilot targeted medicines policies that provide incentives to industry, drug distributors, procurement officers, prescribers, dispensers, and patients, to supply and use the most clinically appropriate medicines that achieve valuable health outcomes in an affordable way. Policy approaches, adopted mostly in high-income countries, [24–27] typically address either cost or quality of care. Pay-for-performance programs that financially reward prescribers for achieving performance metrics related to cost or quality and so-called “value-based” coverage policies that reduce the out-of-pocket cost for medicines known to improve health outcomes combine both dimensions. While evidence is mixed on the effects of pay-for-performance and value-based benefit policies in high-income countries, evidence on the impacts of specific medicines benefit policies in LMIC is virtually non-existent [16].

Given resource limitations, we suggest that most UHC schemes in LMIC consider developing a minimum medicines benefit package that covers a selected list of cost-effective first-line medicines for the most prevalent conditions – many of which are essential medicines [28] available as quality generic products. Given member demand and pressure from providers, schemes also need to decide whether and how to cover innovative, high-cost medicines that have established clinical value but that may benefit fewer patients. Different tools and approaches are needed to develop a minimum medicines benefit and to make decisions about coverage of specialized high-cost medicines; in both cases, it is crucial for the insurance system to monitor utilization, quality of medicines use, and expenditures.

### Key challenges

#### Better information

Information is the single greatest resource that well-functioning UHC systems have at their disposal. However, in many systems, data may only be available about medicines expenditures as a component of overall expenditures, and sometimes even that is unmeasured. Without more granular information about which medicines are used, who prescribes them, how they meet the care needs of specific patients, whether they achieve intended population health outcomes, and whether they are fairly and affordably priced, systems striving toward UHC will find it difficult to ensure clinical quality, efficiency, and safety in medicines coverage.

As UHC-targeting schemes expand, they will need to use electronic payment systems. It is crucial that these systems be designed to include the level of detail required to actively manage a medicines benefit. The Table lists illustrative performance indicators in four main pharmaceutical policy domains (Table 1). Routine information systems should allow measurement of these or similar indicators. However, high-end electronic systems take resources and time to develop. In the meantime, other readily available sources of routine data, such as prescriptions and dispensing records at health facilities, can allow stakeholders to generate information needed to make evidence-informed decisions.

**Table 1:** **Examples of indicators to monitor medicines policies in UHC systems**

Domain and question	Sample indicator	Possible data source
**Availability of quality generic and innovative products**		
Does local industry produce a reliable supply of needed high-quality, low-cost generics?	Volume and percent of locally-produced generics that meet quality and price standards	Industry data
		Quality test records
Do risk-sharing strategies between pharmaceutical companies and payers to provide access to selected high-cost medicines for selected patients achieve intended outcomes, and what are unintended ones?	Percent patients with a target conditions who receive an innovative product that results in expected health benefits	Industry risk sharing program data
**Equitable access**		
Do people receive the medicines they need?	Percentage of people with a diagnosed chronic illness who currently have an appropriate medicine available to treat their condition	Household surveys
How does access to appropriate medicines differ between groups of patients?	Percentage of patients with a given diagnosis who receive a recommended first-line drug in different subgroups (categorized by insurance scheme, geographic location, age group, gender, race and ethnicity, socioeconomic status)	Prescriptions or dispensing records at health facilities
		Insurance claims records
**Appropriate use**		
Are we overusing medicines?	Percentage of primary care patients who receive an antibiotic	Prescriptions or dispensing records at health facilities
		Insurance claims records
Are we underusing medicines?	Percentage of patients with a chronic condition (e.g., diabetes, hypertension) who receive at least one of the recommended treatments	Prescriptions or dispensing records at health facilities
		Insurance claims records
**Affordable costs**		
How much does the insurance scheme/health system spend on medicines?	Medicines expenditures per member per month (overall, by member category, by specific diagnoses, by specific therapeutic categories)	Insurance reimbursement records
What is the financial burden associated with medicines spending in households?	Proportion of patients who indicate that they forego medicines treatment or spend less on other basic needs due to medicines costs	Household surveys

In sum, leaders working toward UHC need to strengthen system capacity in benefit design, information technology, pharmaceutical cost analysis, quality measurement, quality assurance, and interpreting routine longitudinal data on medicines availability, access, use, and expenditures.

### More patient-centered systems

Most LMIC health systems were developed primarily to treat acute conditions and provide inpatient care. The global epidemiologic transition and the increasing need for efficient management of chronic illnesses now dictate that health systems develop ways to provide continuous, affordable, high quality care for life-long illnesses, moving care away from hospitals into the community and taking greater advantage of non-physician providers including nurses, pharmacists, and community health workers. A challenge for UHC-targeting systems will be to link community-based care – such as adherence support at community providers’ offices, pharmacies, and homes - with insurance financing.

Culture is an important determinant of medicines use. LMIC insurance systems need to develop approaches that promote appropriate traditional treatment and avoid inappropriate care – such as using herbal remedies rather than unnecessary antibiotics to soothe symptoms of upper respiratory tract infections. Social marketing efforts will be required to explain both the concept of insurance, often unfamiliar in LMIC, as well as the rationale for specific medicines benefit policies.

Everywhere, decision makers face challenging ethical questions when setting spending priorities in light of resource constraints. Values, population needs, cultural contexts, the overall health care environment, and other economic and social constraints must factor into decisions. Approaches are certain to change over time, as population needs, technologies, and systems change. Transparency in decision making about medicines coverage [21] and accommodating the preferences of civil society in benefit discussions [29] will be key to maintaining public trust.

Health systems, and the broader national and global systems of which they are a part, are complex and dynamic. Interventions by any one actor will impact the behavior of others. Evaluating the impacts of medicines policies in LMIC will require a system perspective [30] to ensure that the intended and unintended effects of policies are known and inform continued system learning.

## Summary

LMIC working toward UHC have enormous potential to improve health. To succeed, they need to adopt an explicit system focus on sound, evidence-informed medicines policies. We highlight four key pharmaceutical system objectives and outline policy options and essential information needed to achieve them.

## Authors’ information

This contribution emerged from decades of efforts by the authors to improve medicines situations in low and middle-income countries. For more than 20 years, AKW and DRD have led research and capacity strengthening activities to contribute to efficient medicines management and sound pharmaceutical policies in systems. Under their Medicines and Insurance Coverage (MedIC) Initiative, they collaborate with health insurance schemes on the path to universal health coverage. JDQ was Director of Essential Drugs and Medicines Policy at the World Health Organization from 1996 to 2004. As President and CEO of Management Sciences for Health, he has guided the first global Dialogue on UHC and Medicines.

## Abbreviations

UHC: Universal health coverage.
